# Metabolism navigates neural cell fate in development, aging and neurodegeneration

**DOI:** 10.1242/dmm.048993

**Published:** 2021-08-04

**Authors:** Larissa Traxler, Jessica Lagerwall, Sophie Eichhorner, Davide Stefanoni, Angelo D'Alessandro, Jerome Mertens

**Affiliations:** 1 Neural Aging Laboratory, Institute of Molecular Biology, CMBI, Leopold-Franzens-University Innsbruck, Tyrol 6020, Austria; 2 Laboratory of Genetics, The Salk Institute for Biological Studies, La Jolla, CA 92037, USA; 3 Department of Biochemistry and Molecular Genetics, University of Colorado Denver, Aurora, CO 80045, USA

**Keywords:** Brain aging, Epigenetics, Metabolic state, Neural development, Psychiatric disorder

## Abstract

An uninterrupted energy supply is critical for the optimal functioning of all our organs, and in this regard the human brain is particularly energy dependent. The study of energy metabolic pathways is a major focus within neuroscience research, which is supported by genetic defects in the oxidative phosphorylation mechanism often contributing towards neurodevelopmental disorders and changes in glucose metabolism presenting as a hallmark feature in age-dependent neurodegenerative disorders. However, as recent studies have illuminated roles of cellular metabolism that span far beyond mere energetics, it would be valuable to first comprehend the physiological involvement of metabolic pathways in neural cell fate and function, and to subsequently reconstruct their impact on diseases of the brain. In this Review, we first discuss recent evidence that implies metabolism as a master regulator of cell identity during neural development. Additionally, we examine the cell type-dependent metabolic states present in the adult brain. As metabolic states have been studied extensively as crucial regulators of malignant transformation in cancer, we reveal how knowledge gained from the field of cancer has aided our understanding in how metabolism likewise controls neural fate determination and stability by directly wiring into the cellular epigenetic landscape. We further summarize research pertaining to the interplay between metabolic alterations and neurodevelopmental and psychiatric disorders, and expose how an improved understanding of metabolic cell fate control might assist in the development of new concepts to combat age-dependent neurodegenerative diseases, particularly Alzheimer's disease.

## Abstract (precis)

**Summary:** Distinct metabolic states regulate the identity of neural cells during development and adulthood. Metabolic alterations due to changes in nutrient availability and aging destabilize cell identity and contribute to neurological diseases.

## Introduction

**Fig. 1.**
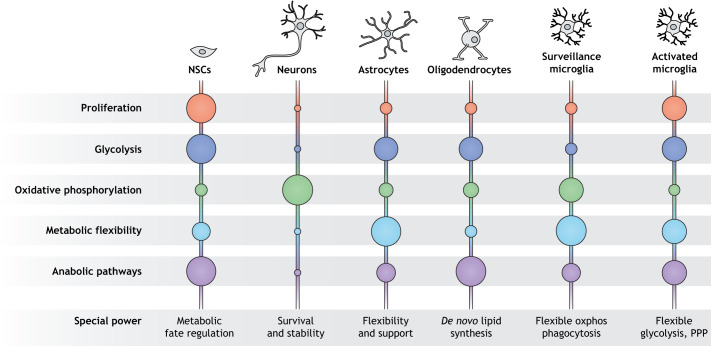
**Metabolic states of brain cell types.** Every brain cell has distinct priorities and metabolic needs, and thus resides in a unique metabolic state that supports its physiological function. Neural stem cells (NSCs) are highly proliferative and depend upon both glycolytic and anabolic pathways. They require a certain degree of metabolic flexibility to allow cell fate regulation to generate neurons, astrocytes or oligodendrocytes. A complete switch to oxidative phosphorylation is a prerequisite for differentiation into neurons, which prioritize survival and stability. Astrocytes provide metabolic support for neurons and thus stand out with their immense metabolic flexibility. They can switch to alternative carbon sources in a hypoglycemic state and generate an excess of lactate through glycolysis, which can be used by neurons to fuel oxidative phosphorylation. Oligodendrocytes are characterized by the production of lipid-rich myelin to cover axons for more effective signal transduction. They thus use a great proportion of their resources to support anabolic pathways for *de novo* lipid synthesis. By contrast, microglia do not stem from NSCs, but migrate to the brain during development. In their neuroprotective surveillance state, they mainly rely on oxidative phosphorylation and are highly flexible to adapt to changes in glucose and oxygen availability. In their activated state, microglia start proliferating and switch to glycolysis as their main energy source. PPP, pentose phosphate pathway.

Cell identity and function are inseparable from a cell's metabolism of energy and molecular components. The dominance of specific metabolic pathways, reaction chains and individual metabolites collectively determine the cellular metabolome and delineate distinct metabolic states in which certain cell types can reside. Metabolic states have a direct impact on epigenetic control of cell function and fate, and may promote certain epigenetic states, while suppressing others (Wong et al., 2017). Each cell type of the brain possesses a unique metabolic profile, and to ensure fate stability and sustained cellular functionality, only a limited degree of metabolic flexibility is permitted to adapt to external cues (Fig. 1). It is becoming increasingly evident that the metabolic state of the cell is consequential beyond the mere adaptation of energetic or anabolic needs, and is a master regulator of many signaling pathways, transcription factors and epigenetic remodelers, all of which regulate cell fate changes or maintenance during development (Enzo et al., 2015; Tran et al., 2019). During neural development, the constant metabolic rewiring of neurodevelopmental stem and precursor cells is crucial to regulate stemness, proliferation, differentiation, apoptosis, maturation and integration. Several individual metabolites have been identified that directly control the epigenetic landscape and chromatin rearrangements, thus permitting targeted differentiation of precursor cells. It is therefore unsurprising that mutations in metabolic enzymes are a common cause of congenital brain abnormalities (Ashe et al., 2019; Lake et al., 2015). Mature neurons, contrary to stem cells and glial cells, prioritize network maintenance over regeneration. They consequently rely on a highly specialized metabolic state that strictly protects neuronal cell fate and ensures the most efficient energy generation (Bélanger et al., 2011; Houck et al., 2018). Similar to the deterministic role of metabolism during development, certain metabolic states can safeguard or destabilize the cell identity of differentiated neurons and glial cells. Metabolic changes that go beyond a sustainable level of metabolic flexibility thus challenge cellular resilience and brain function, and have shifted to the forefront of aging and neurodegeneration research.

In this Review, we will first provide a framework for the role of metabolism in neural fate determination during neural development, and for neural cell function and fate stability in the adult brain. We will subsequently reflect upon current concepts concerning the involvement of metabolic states and individual metabolites in the control of the epigenetic landscape and cell identity. This will be complemented by integrating the advanced knowledge garnered in the cancer research field. Further, we will highlight the metabolic contributions to genetically driven neurodevelopmental diseases, metabolic signatures of psychiatric disorders, and the potential roles of metabolic states in aging and sporadic neurodegenerative diseases.

## Physiological roles of metabolism: effects beyond energy production

The development of the nervous system is characterized by the extensive proliferation of neural stem and progenitor cells (NSCs) and sequential fate decisions that depend on precise patterning cues to permit proper regionalization, differentiation and functional integration of new cells (Rifes et al., 2020; Wen et al., 2009). Highly proliferative cells require building blocks for organelles, DNA and membranes, and thus depend on a high glycolytic activity to support the anabolic needs of proliferation (Candelario et al., 2013). At the same time, NSCs also need to make sequential decisions about which cell fate they will acquire next. During the differentiation of multipotent NSCs into further differentiated cell types, metabolic adaptation needs to occur. Specifically, this adaptation comprises a shift from supporting cells of a highly proliferative nature towards more restricted NSC types, and eventually to somatic cells including post-mitotic fates. Metabolic pathways and individual metabolites play key roles in cell fate determination that reach far beyond their roles in mere adaption to anabolic processes. Energy demands that distinguish proliferative from post-mitotic cells, as well as mature neurons, astrocytes and oligodendrocytes, all have very different priorities and requirements, and therefore reside in different metabolic states.

### Metabolic regulation of neural development

Embryonic NSCs are highly metabolically active and thus share epigenetic, transcriptomic and metabolic signatures with other highly proliferative cells during development and with some cancer cell types (Box 1). Yet, NSCs stand out by their metabolic flexibility, which allows them to metabolize carbohydrates, fatty acids or amino acids to balance catabolism and anabolism to their current needs (Folmes et al., 2012). During the early stages of NSC development, glycolysis and shunting of its intermediates through the pentose phosphate pathway fuels anabolism (Fig. 1) (Candelario et al., 2013; Folmes et al., 2012; Sá et al., 2017; Stincone et al., 2015). Glycolysis is the most ancient strategy for cells to generate energy in the form of adenosine triphosphate (ATP) from glucose and other sugars. Additionally, glycolysis provides intermediates for various anabolic pathways, such as the biosynthesis of serine or reducing factors, or the pentose phosphate pathway, which produces the nucleotide precursors required to maintain rapid proliferation and survival. Disturbances in glucose metabolism during neural tube fate determination critically affect embryogenesis. Maternal diabetes can lead to severe developmental defects, such as anencephaly, a condition in which infants lack major parts of their brain, but can also cause milder phenotypes, including learning deficiencies or autistic traits (Dheen et al., 2009; Márquez-Valadez et al., 2018; Xie et al., 2016). Such changes in glucose availability can affect the fine balance of proliferation and differentiation during neural development, which is mediated by the inhibitory effects of excessive glucose concentrations on c-Abl (also known as Abl1)/p53 (also known as Tp53)-mediated apoptosis or through epigenetic alterations, such as elevated histone acetylation, and consequential premature differentiation (Fu et al., 2006; Ji et al., 2019; Jia et al., 2008; Liu et al., 2011).

**Box 1. The Warburg effect and neurodegeneration**

The Warburg effect was initially described as the metabolic switch to aerobic glycolysis despite the presence of oxygen during malignant transformation in cancer (Warburg, 1956). It is now known that the switch to the energetically inefficient glycolytic pathway does not only aim for generating anabolic precursors to drive proliferation, but rather represents a pathological program initiating de-differentiation and loss of cell identity (Intlekofer and Finley, 2019). Contrary to initial hypotheses, decreased oxidative phosphorylation in cancer cells is due to a repurposing of metabolic intermediates of the TCA cycle. The accumulation of mitochondrial metabolites often induces malignant transformation through epigenetic mechanisms, enhancing survival and proliferation independent of nutrient availability (Lee et al., 2014). For example, the oncometabolite 2-hydroxyglutarate (2-HG) increases methylation patterns, or the accumulation of acetylCoA leads to the acetylation of histones to specifically promote tumor proliferation (Fig. 3) (Cai et al., 2011; Schvartzman et al., 2019). Rewiring of metabolism is further associated with changes in metabolic gene expression and splicing. Tumor cells often express high levels of the glycolytic enzymes hexokinase 2 (HK2) and 6-phosphofructo-2-kinase/fructose-2,6-biphosphatase 3 (PFKFB3), or switch isoforms of metabolic enzymes such as PKM (Alves-Filho and Pålsson-McDermott, 2016; Lincet and Icard, 2015). Post-mortem brain analysis revealed some interesting similarities of glucose metabolism between cancer and neurodegeneration (Marinaro et al., 2020 preprint). A Warburg-like metabolic switch in brain cells, including neurons, may further cause neural cell de-differentiation and loss of cell fate stability, leading to loss of functionality (synaptic transmission) and, ultimately, cell death (Mertens et al., 2021). In contrast to most somatic cells, neurons developed unique mechanisms to maintain cellular integrity, which might explain their opposite response to Warburg-like changes. PPP, pentose phosphate pathway.
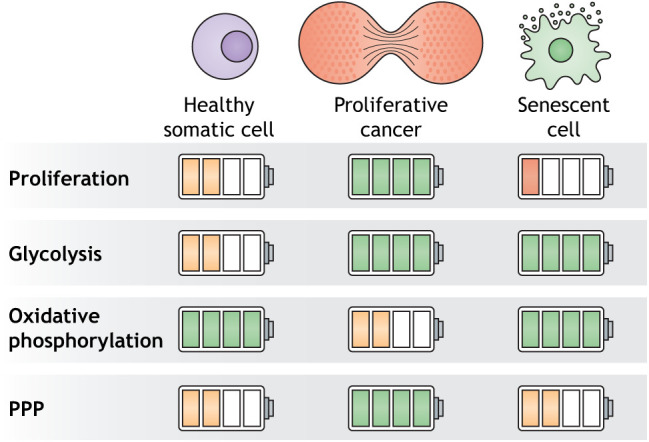

By definition, all stem cells have the ability to undergo symmetric and asymmetric cell division (Smith, 2006). Symmetric division leads to an expansion of the stem cell pool, whereas the net effect of asymmetric division is that it leads to more differentiation towards lower-hierarchy stem and precursor cells, neurons and glia (Gómez-López et al., 2014). Divergent stem cell fates are partly fueled by their metabolic flexibility to prioritize cell type-specific metabolic programs to adapt to energetic needs and to control genetic programs of the evolving cell fate (Folmes et al., 2012). The most dramatic example for the fate-determining power of metabolic states is the metabolic switch from glycolysis to oxidative phosphorylation during the differentiation of NSCs to neurons (Candelario et al., 2013; Zheng et al., 2016). Interestingly, the future cell identity is already determined in the precursor state, and a smooth transition to oxidative phosphorylation in NSCs is a prerequisite for neuronal differentiation, whereas preservation of glycolysis induces a glial fate (Lorenz and Prigione, 2017). As such, forced activation of glycolysis by overexpression of hexokinase 2 (HK2) or lactate dehydrogenase (LDHA) during *in vitro* differentiation of human induced pluripotent stem cell (iPSC)-derived NSCs blocks neuronal differentiation and promotes cell death and astrocyte fate (Zheng et al., 2016). The metabolic intermediates α-ketoglutarate (αKG) and acetylCoA moderate the switch from symmetric to asymmetric division in a metabolic state- and rate-dependent manner through the activation of neuronal or glial genes (Balasubramaniyan et al., 2006; Carey et al., 2015; Zhou et al., 2019). Failure of late-stage NSCs to switch from high glycolytic activity to αKG and acetylCoA production results in the blockade of NSC differentiation (Luo et al., 2013; Majumder et al., 2013; Zhou et al., 2019).

Neural crest cells are a neuronal stem cell population that form the peripheral nervous system, including sensory neurons, cartilage and melanocytes (Minoux et al., 2010). These cells represent a migratory stem cell population, which delaminate from the neural tube to undergo an epithelial-to-mesenchymal transition (EMT) (Bhattacharya et al., 2020). Pronounced activation of glycolysis is a requirement for neural crest EMT and migration, and it was shown that this phenomenon is linked to the EMT-promoting YAP/TEAD-mediated Hippo signaling pathway. Inhibition of glycolysis in neural crest cells blocks the interaction of Yes-associated protein 1 (YAP1) with transcription enhancer factor (TEAD) and thus prevents EMT and neural crest migration (Bhattacharya et al., 2020).

All these observations exemplify how metabolism controls sequential cell fate changes during neuronal development. Metabolites control gene expression on the epigenetic and signaling pathway levels in a highly concerted manner and thus challenge the conventional view whereby metabolic changes are solely an adaption to changes in energy demands. Often, the fate-determining activity of metabolism is associated with distinct metabolic states and switches between these states are imperative.

### Metabolic states of adult brain cells

The function of the brain as a complex cellular network relies on several layers of structural and cellular interactions, as well as on the global and local metabolic collaboration between specialized cell types. Despite making up for only 2% of the body weight, the adult human brain consumes around 20% of available oxygen and 25% of available glucose (Raichle and Gusnard, 2002). Neurons alone require vast amounts of energy to sustain the ion gradients required to maintain an excitable resting potential and to generate action potentials (Mergenthaler et al., 2013). To meet those energy demands, neurons rely heavily on oxidative phosphorylation in mitochondria, with a negligible proportion of their energy stemming from glycolysis (Fig. 1) (Itoh et al., 2003; Zhang et al., 2014; Zheng et al., 2016). In contrast to glycolysis, which is among the most ancient metabolic pathways that occur in virtually all organisms, oxidative phosphorylation instead depends on mitochondria, which are where the respiratory chain and ATP synthase are operating. Per molecule of oxidized glucose, oxidative phosphorylation yields 30 molecules of ATP, rendering it 15-fold more efficient than glycolysis (Alberts et al., 2002). On the downside, neuronal oxidative phosphorylation inevitably engenders reactive oxygen species (ROS) and appropriate countermeasures; further, it strictly requires oxygen to function. This makes neurons the biggest oxygen consumers in the brain (Bélanger et al., 2011). Immature, newborn neurons, similar to NSCs, respond to the inhibition of glycolysis with cell death, whereas mature neurons are indifferent to glycolysis inhibition, highlighting their exclusive reliance on oxidative phosphorylation (Candelario et al., 2013). Strikingly, mature neurons even lack key glycolytic enzymes, such as the 6-phosphofructo-2-kinase/fructose-2,6-biphosphatase 3 (PFKFB3), which limits a neuron's ability to activate glycolysis in response to bursts of energy demands (Bolaños et al., 2010; Herrero-Mendez et al., 2009).

Why do neurons forgo glycolysis, even at times of acute need? One potential answer supported by experimental data is that glycolysis, or certain glycolytic metabolites, are incompatible with neuronal identity and survival. For instance, overexpression of PFKFB3 in neurons leads to extensive oxidative stress and cell death (Bolaños et al., 2010; Herrero-Mendez et al., 2009; Liot et al., 2009). Interestingly, inhibition of PFKFB3 showed neuroprotective effects in a mouse model of cerebral ischemia, further supporting the idea that activating glycolysis has negative effects in mature neurons (Burmistrova et al., 2019).

To explain how neurons respond to energy bursts during synaptic activation or changes in nutrient availability, we have to look at the whole picture in the brain. In direct comparison to neurons, glial cells are metabolically flexible, which enables them to respond to environmental changes relating to nutrient availability (Fig. 1) (Bélanger et al., 2011). Astrocytes upregulate glycolysis in response to mitochondrial dysfunctions and can even adapt to sustained hypoglycemia by activating pathways to metabolize alternative carbon sources, such as glutamate, aspartate or alanine (Almeida et al., 2004; Weightman Potter et al., 2019). Thus, during periods of nutrient scarcity or during energy-demanding processes like synaptic bursts, astrocytes fuel neurons with metabolic intermediates to sustain their function (Chuquet et al., 2010; Sá et al., 2017). The most prominent support strategy in this regard is the so-called astrocyte–neuron lactate shuttle, by which astrocytes produce and release lactate. Neurons take up lactate from their astrocyte neighbors via monocarboxylate transporters (MCTs) and express the lactate dehydrogenase B isoform (LDHB), which converts lactate back to pyruvate to be fed into the tricarboxylic acid (TCA) cycle (Mason, 2017). In addition to supporting neurons with metabolic precursors, astrocytes clear the synaptic cleft of neurotransmitters remaining from synapse transmission activity (Oliet et al., 2001). This function is critical to maintain synaptic and tissue homeostasis, as excess local levels of GABA or glutamate have neurotoxic properties (Mahmoud et al., 2019). Following uptake, astrocytes convert both GABA and glutamate neurotransmitters to glutamine. This recycled product is then shuttled back to neurons, where it is used as a precursor to re-fill neurotransmitter vesicles, or alternatively for oxidative phosphorylation via the TCA cycle (Bak et al., 2006). This close metabolic relationship between neurons and astrocytes provides a strong framework for a stable metabolic environment in the brain.

Beyond their neuron-supporting roles, astrocytes are key regulators of neuroinflammation due to their tight interaction with microglia (Haim and Rowitch, 2017; Liddelow et al., 2017). Microglia are the resident immune cells of the brain; they survey the brain for damage to clear debris and support neuronal homeostasis through the release of trophic factors. Microglia mainly rely on oxidative phosphorylation in this surveillance state, but remain metabolically flexible (Fig. 1). This metabolic flexibility of microglia is essential to maintain brain homeostasis in periods of limited glucose or oxygen availability (Bernier et al., 2020; Lauro and Limatola, 2020). When exposed to inflammatory stimuli, microglia switch to the pro-inflammatory, activated state, which induces reactive astrocytes and neuronal demyelination through the secretion of cytokines (Domingues et al., 2016; Liddelow et al., 2017). The activation of microglia is associated with a metabolic switch from oxidative phosphorylation to glycolysis, similar to what is described in peripheral immune cells (Bernier et al., 2020; Lauro and Limatola, 2020). Notably, extracellular levels of the metabolic intermediates succinate or lactate further modulate microglia activity (Liu et al., 2020; Peruzzotti-Jametti et al., 2018).

Oligodendrocytes are a type of glial cell that is essential for the propagation of axonal signals in neurons, as they wrap neuronal axons to cover them with an insulating myelin sheath. During differentiation and the myelination process, oligodendrocyte precursor cells require huge amounts of energy and fatty acids, the main component of myelin (Fig. 1) (Dimas et al., 2019; Fünfschilling et al., 2012). Thus, they are extremely vulnerable to energy deprivation and hypoglycemia, and require metabolic intermediates from astrocytes for proper function (Camargo et al., 2017; Rosko et al., 2019). After the energy-demanding myelination during development, a metabolic switch to aerobic glycolysis marks oligodendrocyte maturation. In the post-myelination phase, oligodendrocytes supply lactate directly to neuronal axons, providing metabolic support in a similar manner to astrocytes (Philips and Rothstein, 2017).

These examples show that distinct metabolic states support the unique function of each cell type in the brain. Neurons possess the most specialized metabolism in the brain, and depend almost exclusively on oxidative phosphorylation. Metabolically flexible glial cells, such as astrocytes and oligodendrocytes, support neurons during periods of high energy demands or nutrient scarcity by providing lactate as an alternative carbon source. Neurons, astrocytes and oligodendrocytes have established a metabolic interaction to provide a stable microenvironment in the brain, which is supported by microglia secreting neurotrophic factors. Further, metabolites function as signals and the accumulation of certain metabolites can trigger an activation of microglia, which can subsequently lead to an inflammatory and neurotoxic environment.

## Epigenetic landscaping in metabolism's backyard

Recently, there has been marked excitement regarding the molecular epigenetic mechanisms that execute metabolic control over cell fate (Lu et al., 2012; Ward and Thompson, 2012). Mutated metabolic enzymes and aberrantly accumulated metabolites have been reported to affect the epigenetic landscape and re-wire cell identity during development, in adulthood and in disease (Box 1) (Reid et al., 2017). Here, we highlight some of the histone and DNA modifications that have been studied extensively and are thus well understood, and discuss their relation to metabolism.

Cell fate specification and the maintenance of cell identity are established by highly specific epigenetic control of transcription. On the one hand, the epigenetic landscape of a cell requires stability as it safeguards cell identity; on the other hand, it also needs a certain degree of plasticity to allow cells to adapt to environmental cues such as hypoxia, limited nutrient availability or surges in energy demand. The regulation of the epigenetic landscape is accomplished by chromatin-modifying enzymes, and their activity depends directly on the availability of specific metabolites (Etchegaray and Mostoslavsky, 2016; Li et al., 2020; Lu et al., 2012). In this way, metabolism acts as the mediator between cellular needs and cellular adaptations.

**Fig. 2.**
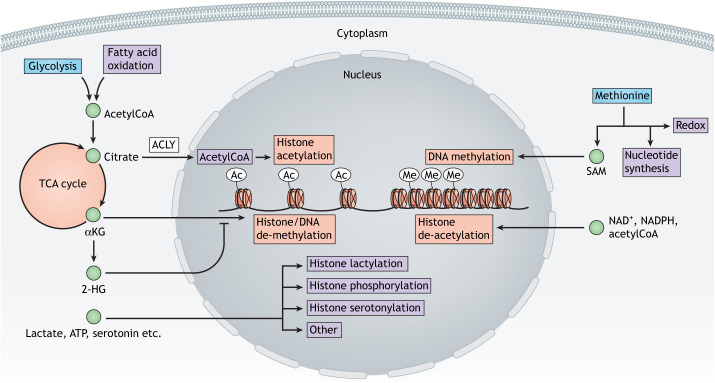
**Metabolic control mechanisms over cell fate and function.** Metabolic intermediates affect cell fate specification through specific epigenetic control of transcription. AcetylCoA is at the intersection of glycolysis and fatty acid oxidation, and thus represents the metabolic state of the cell. Adenosine triphosphate (ATP) citrate lyase (ACLY) generates acetylCoA from citrate in the nucleus to increase histone acetylation to open up chromatin for active gene transcription. Citrate can be metabolized further to α-ketoglutarate (αKG) in the tricarboxylic acid (TCA) cycle in mitochondria, and αKG acts as a cofactor for histone and DNA demethylases to further control chromatin openness. Increased metabolism of αKG to 2-hydroxyglutarate (2-HG) inhibits αKG-dependent de-methylation of DNA and histones. Other metabolites, such as lactate, ATP or serotonin, can also activate gene transcription through histone lactylation, phosphorylation or serotonylation. By contrast, methionine metabolism generally inhibits gene transcription through S-adenosylmethionine (SAM), the methyl donor for histone and DNA methylation. NAD^+^, NADPH and acetylCoA additionally activate histone de-acetylation to compact chromatin and inhibit gene transcription. A highly orchestrated interaction of methylation and acetylation, and thus tight metabolic control, is required for cell fate control. Ac, acetylation; Me, methylation.

AcetylCoA is a key metabolic intermediate and is regarded as highly representative of the nutritional state of a cell because it functions at the intersection of canonical glycolysis, TCA cycle and fatty acid metabolism (Fig. 2) (Shi and Tu, 2015). High glucose levels directly reflect on the acetylCoA:CoA ratio, which regulates histone acetyltransferase (HAT) activity, contributing to increased chromatin accessibility and gene activation (Lee et al., 2014). The HAT counterparts histone deacetylases (HDACs), which contribute to gene silencing by removing acetylation marks from histones, are mainly regulated by the availability of NAD^+^ and NADPH (Fig. 2) (Schlachetzki et al., 2020; Vogelauer et al., 2012). A decrease in NAD^+^ levels accompanied by an increase in acetylCoA levels leads to an imbalance of HAT and HDAC activity, which contributes to reduced transcriptional precision in aging and age-associated disease (Covarrubias et al., 2021; Peleg et al., 2016).

Another fundamental form of epigenetic control that is heavily influenced by metabolism is the methylation of histone proteins and DNA. Similarly to histone acetylation, methylation reactions are regulated by the balance of methyltransferases and demethylases. The activity of these enzymes is dependent on S-adenosylmethionine (SAM) as a substrate for methyltransferases and both the early TCA cycle intermediate αKG and oxygen as co-substrates for demethylases (Fig. 2) (Su et al., 2016). The late TCA intermediates succinate, fumarate and 2-hydroxyglytarate (2-HG) inhibit αKG-dependent demethylases, and thus also control the epigenetic landscape and gene expression (Xiao et al., 2012; Xu et al., 2011; Yong et al., 2020). Notably, an accumulation of these TCA intermediates blocks differentiation in stem cells by mediating H3K9 hypermethylation (indicative of gene silencing and heterochromatin), attributing cell fate control to metabolic alterations (Box 1) (Schvartzman et al., 2019).

Metabolites further regulate several other epigenetic modifications to modulate cell fate (Fig. 2). Lactate, which has long been considered a by-product of glycolysis, directly activates gene transcription by histone lactylation (Zhang et al., 2019). Serotonergic neurons have developed serotonylation of histone 3 to regulate differentiation by activating neuronal genes specifically for the serotonergic subtype (Farrelly et al., 2019). Glucose, amino acid, fatty acid and nucleotide metabolism jointly regulate histone O-GlcNAcylation, a process paramount for the repression of homeostatic genes during development, proliferation and differentiation. By contrast, an accessible chromatin state can be induced by different histone acylations, such as crotonylation, propionylation or butyrylation (Li et al., 2018). Poly(ADP-ribose) polymerase 1 (PARP1)-mediated histone poly(ADP)ribosylation, called PARylation, has recently gained attention in the aging field. PARP1 is an NAD^+^-dependent enzyme that is responsible for chromatin accessibility during DNA repair. Increased oxidative stress and DNA damage in aging lead to increased PARylation and a decreased NAD^+^ pool (Fang et al., 2016). Interestingly, metabolic enzymes that translocate to the nucleus have been shown to affect gene expression, the most prominent example being PKM, which directly phosphorylates histone 3, leading to the expression of cell-cycle and metabolic genes (Alves-Filho and Pålsson-McDermott, 2016; Li et al., 2018).

## Deterministic roles of metabolic alterations in neurological diseases

In the previous sections, we highlighted the role of metabolism in cell fate decisions during neuronal development and in determining cell fate stability and function. The extreme metabolic specialization of neurons and their dependence on a supportive and surveilling network of glial cells renders the brain vulnerable to disease. Here, we will highlight metabolic elements contributing towards and promoting neurodevelopmental, neuropsychiatric and neurodegenerative disorders.

### Neurodevelopmental diseases and metabolism

Many neurodevelopmental disorders are mono- or multigenic disorders. Monogenic disorders can be caused by mutations in a single metabolic enzyme and often show severe neurological phenotypes. The metabolic switch from glycolysis to oxidative phosphorylation is an essential step in neuronal maturation, and lies at the core of many neurodevelopmental disorders. The most common monogenic disorders are Leigh syndrome (LS) and mitochondrial encephalopathy lactic acidosis and stroke-like episodes (MELAS) (El-Hattab et al., 2015; Lake et al., 2015). They are caused by single mutations affecting electron transport complex components, such as NDUFS1, and mitochondrial transfer RNAs (tRNAs), such as m.3243A>G in tRNA^Leu(UUR)^ (El-Hattab et al., 2015; Lake et al., 2015). Both syndromes are characterized by severe metabolic dysfunctions, as indicated by ATP depletion and the upregulation of glycolysis as a compensatory mechanism, which results in the accumulation of lactate in brain tissue and the cerebrospinal fluid (CSF) (Iannetti et al., 2018; Lake et al., 2015). Brain regions with high energy demands, such as the brainstem or basal ganglia, are most vulnerable to ATP depletion during development. The severity of such mitochondrial diseases always depends on the number of affected mitochondria, as mutations in mitochondrial DNA usually occur in only a fraction of all mitochondria present in a cell, also termed heteroplasmy. Depending on the percentage of heteroplasmy of mitochondria carrying the causative mutation, the disorder leads to a corresponding increase of glycolysis, resulting in hyperlacticacidemia (Iannetti et al., 2018).

NSCs carrying complex I mutations have a decreased ability to differentiate towards neurons, and this instead favors glial fates (Cabello-Rivera et al., 2019; Zheng et al., 2016). During the early stages of neural development, oxidative phosphorylation only plays a minor role, and compensatory mechanisms enable neurons to develop. These newborn neurons are immature and characterized by reduced mature neuronal gene expression that is associated with neuronal differentiation, neuronal signaling and synaptic processes, as well as a temporarily increased expression of immaturity markers, such as doublecortin (Johnson et al., 2016). Even though a residual part of oxidative phosphorylation activity remains in LS patients with complex I deficiencies, glycolysis is increasingly used to compensate for the defects in oxidative phosphorylation. This might lead to a general instability of neuronal cell identity and a block of maturation. Consequently, these neurons are locked in an immature state and thus more prone to programmed cell death, a feature that is exclusive for precursor cells and immature neurons (Galera-Monge et al., 2020; Kole et al., 2013). Taken together, oxidative phosphorylation deficiencies are often compensated by residual mitochondrial function, but, in conditions with severe oxidative phosphorylation impairments, increased glycolytic activity hinders the development of mature neurons. The lack of maturity results in dysfunctional synaptic transmission and neurodegeneration in the first years of life.

Mutations in metabolic pathways can also lead to an accumulation of metabolites, triggering toxic effects. Neurometabolic disorders are either caused by increased production or by decreased clearance of certain metabolites (Karimzadeh, 2015). A well-described example of neurometabolic disorders is phenylketonuria, which is caused by a mutation in a phenylalanine-degrading enzyme. Accumulation of phenylalanine in the blood is especially toxic to the brain, resulting in severe intellectual disability and psychiatric disorders, which can be avoided by a special diet containing limited phenylalanine (Ashe et al., 2019). Similarly, the accumulation of the oncometabolite 2-HG leads to neurodevelopmental delay and macrocephaly through regulating methylation reactions, as described above (Kranendijk et al., 2012; Xu et al., 2011). Such oncogenic signals further result in increased proliferation and decreased differentiation during NSC differentiation (Moroni et al., 2004; Narbonne-Reveau et al., 2016; Schvartzman et al., 2019). Notably, 2-HG accumulation in post-mitotic neurons leads to aberrant cell cycle activation, cell death and neurodegeneration (Lee et al., 2009; Tommasini-Ghelfi et al., 2019).

In summary, congenital metabolic defects can lead to a variety of phenotypes. However, they primarily affect the brain and lead to severe neurodevelopmental disorders due to the complex and specialized metabolic states that constitute brain development. Mitochondrial diseases are associated with severe disease progression and are often lethal in early life. This is due to the compensation of oxidative phosphorylation with glycolysis leading to cell fate instability and thus cell death. By contrast, neurometabolic disorders caused by a toxic accumulation of metabolic intermediates can often be treated by changes in diet; however, when left untreated, they can confer serious toxicity to the brain.

### Psychiatric disorders and metabolism

Psychiatric disorders have a considerable environmental component that dwarfs the effects of genetic factors, even when high-seeming heritability scores are proposed (Moore and Shenk, 2017; Pettersson et al., 2019). Disorders such as autism spectrum disorder (ASD), schizophrenia, major depressive disorder or bipolar disorder (BD) are therefore biologically and clinically extremely heterogeneous. Metabolic alterations during pregnancy, such as intrauterine hyperglycemia, increase the risk for developing ASD and other psychiatric diseases (Hoirisch-Clapauch and Nardi, 2019). Gestational diabetes not only triggers oxidative stress, but directly affects the epigenome and signaling cascades regulating neuronal connectivity and neuronal guidance during development. Alterations in folate availability during development further affect the balance of proliferation, differentiation and cell death in the brain, which is a hallmark of ASD (Melnyk et al., 2012). Folate is an essential component of the one-carbon metabolism, regulating nucleotide biosynthesis, DNA methylation and glutathione synthesis, thus affecting several pathways from epigenetic regulation to buffering oxidative stress (Melnyk et al., 2012). Indeed, post-mortem brain analysis of ASD patients showed reduced SAM levels associated with a general hypomethylation, decreased glutathione levels associated with increased oxidative stress, and decreased purine and pyrimidine levels associated with aberrant proliferation (Melnyk et al., 2012; Orozco et al., 2019). Interestingly, similar metabolic changes affecting the epigenome and oxidative stress regulation occur in several other neuropsychiatric disorders, such as Down syndrome or schizophrenia (Krebs et al., 2009; Obeid et al., 2012; Orozco et al., 2019). Opposing results throughout studies, however, did not allow for the development of treatment strategies targeting one-carbon metabolism in ASD patients (Main et al., 2010).

Modeling psychiatric disorders has proven difficult, and it is thus equally challenging to diagnose and find proper treatments for patients suffering from these disorders. Most psychiatric disorders are currently diagnosed using rather subjective clinical questioning, which can bias the allocation of patients to specific subgroups or even diseases (Smith et al., 2020; Yang et al., 2013). Metabolic phenotyping can help to stratify different subpopulations of patients with psychiatric disorders to categorize them for targeted treatment approaches (Hagihara et al., 2019; Smith et al., 2020). Metabolites in blood serum or urine have been proposed as relatively reliable early biomarkers for the diagnosis and treatment success of neuropsychiatric disorders, such as autism (Bent et al., 2018; Emond et al., 2013; García-Gutiérrez et al., 2020; Smith et al., 2020). Unfortunately, it is often difficult to infer molecular disease mechanisms from metabolic studies that are based on biomarker identification in blood or urine. Compounding this issue, the scarcity of adequate human model systems to study neuropsychiatric disorders remains a fundamental challenge. Post-mortem studies of the brain have provided useful insight into these disorders. One major limitation is that there are usually decades between the onset of a neuropsychiatric disorder and the analysis of brain metabolomics (Lan et al., 2009; Yoshimi et al., 2016). Additional changes accumulate in the brain during that period, which are not necessarily associated with the disease, and might disguise the actual disease mechanism. Animal models often do not fully recapitulate the phenotypic hallmarks of neuropsychiatric disorders, and are thus not utilized in many metabolic studies. *In vitro* generation of patient-specific neurons, astrocytes or microglia from iPSCs represents a model to assist in the improved understanding of early changes in brain disorders. In this regard, hippocampal neurons differentiated from BD patient iPSCs display a profound mitochondrial hyperactivity that correlates with the pathologic hyperexcitability of the neurons (Mertens et al., 2015). Despite this serving as an example of how iPSC-based studies could provide insight into the intertwining of metabolic states with disease phenotypes, and clear evidence on a metabolic involvement in key neuronal disease features, several questions remain unsolved. Further studies are needed to pinpoint excess oxidative phosphorylation as either a cause or consequence of excitability changes in diseased neurons. NSCs carrying a mutation in the autism risk gene, tri-methyl-lysine hydroxylase (*TMLHE*), were shown to have a reduction in fatty acid oxidation during the early stages of neuronal development (Xie et al., 2016). This disturbs the balance of symmetric and asymmetric divisions of NSCs and leads to extensive symmetric differentiation and an exhaustion of the stem cell pool (Xie et al., 2016). This pre-mature differentiation of neurons is characteristic for ASD and often results in macrocephaly, most probably due to the shorter period of apoptosis competence during development (Schafer et al., 2019).

Metabolic alterations have been described in most neuropsychiatric disorders, and metabolite studies have prompted an optimistic perspective towards the identification of new biomarker signatures in blood or urine to aid diagnosis. Patient-based iPSC-based models might provide a platform to study mechanistic contributions of metabolism to psychiatric disorders in neural cell models in the future (Santos et al., 2021).

### Brain aging and metabolism

Age-associated diseases are on a trajectory to become a monumental burden for our societies. Accumulation of cellular damage throughout life causes a variety of diseases: both cancer and neurodegeneration are equally dramatic examples. Neurons are long-lived cells that persist throughout a lifetime, yet they are still prone to aging and consequently accumulate damage. They require extraordinary mechanisms to safeguard neuronal cell identity despite the accumulated damage, which protects them from cell death and malignant transformation (Houck et al., 2018; Kole et al., 2013). Interestingly, mature neurons virtually never regress into malignant tumor cells, but instead are susceptible to age-associated dysfunction and degeneration. In this section, we will discuss the metabolic contribution to aging and longevity.

It is becoming increasingly clear that the epigenome most conveniently reflects the biological age of an organism. The epigenetic landscape integrates environmental factors that have accumulated over a lifetime. In contrast to chronological aging, age-associated epigenetic changes lead to transcriptional dysregulation and lesser defined transcriptional profiles. This phenomenon, often referred to as epigenetic drift, is a hallmark of biological aging and can be accelerated or slowed down by drugs and lifestyle, also affecting the risk and onset of age-associated diseases (Fahy et al., 2019; Teschendorff et al., 2013). Metabolism, as a sensor of nutrient availability and stressors, translates environmental signals into epigenetic signatures (Kaelin and McKnight, 2013). Further, most age-dependent metabolic changes, such as dysfunctions in SAM metabolism, have been shown to impact, and thus be reflected, in DNA methylation patterns (Fig. 2) (Parkhitko et al., 2016). The most dramatic example of the power of DNA methylation as a mirror of chronological and biological aging are the so-called epigenetic clocks, which calculate age based on DNA methylation patterns (Horvath, 2013; Levine et al., 2018). These clocks provide a measurable factor of aging and incorporate environmental changes through metabolic regulators. To this end, different clocks exist that are each optimized to measure specific types of age-related changes, such as biological age across human tissues, exact human chronological age, biological fitness or even time to death (Horvath and Raj, 2018). As a result of such precise and convenient readouts being available, several putative anti-aging drugs, often inspired by longevity experiments in *Caenorhabditis elegans* and mice, are being assessed towards their ability to ameliorate aging. Interestingly, such drugs often work through the modification of metabolite availability and activation of metabolic signaling pathways. Rapamycin, a US Food and Drug Administration (FDA)-approved drug, has been shown to extend life span in *C. elegans*, *Drosophila melanogaster* and other model organisms via inhibition of the mechanistic target of rapamycin (mTOR) pathway. The extension of life span also results in an extension of health span by improving age-associated defects, such as cognitive impairment and amyloid beta pathology in transgenic mouse models for genetic variants of familial Alzheimer's disease (AD) (Dai et al., 2014; Majumder et al., 2012; Robida-Stubbs et al., 2012; Spilman et al., 2010). Another anti-aging and diabetes drug, metformin, primarily targets mitochondrial respiration, but also mediates longevity by regulating insulin-like growth factor (IGF-1) and AMP-activated protein kinase (AMPK; also known as PRKAA2) signaling (Menendez, 2020). Interestingly, metformin specifically targets metabolites that affect the epigenetic landscape, such as NAD^+^, acetylCoA, SAM or αKG, thus preventing age-associated epigenetic drift (Menendez, 2020). Remarkably, a study in humans showed that metformin treatment over a year decreases biological age by 2.5 years (Fahy et al., 2019). Additionally, metformin prevents pathological de-differentiation and associated senescence by stabilizing the epigenetic landscape (Fahy et al., 2019; Menendez, 2020; Vazquez-Martin et al., 2012). NAD^+^ supplementation has moved further toward the center of aging research due to its high tolerability. This cofactor is important for redox reactions, but also regulates the activity of sirtuins, CD38 and poly(ADP-ribose)polymerases. Increased DNA damage in aged neurons requires high levels of NAD^+^ for DNA repair mechanisms, leading to the NAD^+^ ablation characteristic of aging (Fang et al., 2016). The lack of NAD^+^ as a cofactor for other enzymes, such as the sirtuin deacetylase family, results in aberrant histone acetylation and loss of cell fate stability. The role of NAD^+^ in epigenetic control, mitochondrial function, cellular senescence, DNA damage response and proteostasis makes it a promising anti-aging intervention – one that is already available as dietary supplement (Aman et al., 2018; Lautrup et al., 2019; Rajman et al., 2018).

**Fig. 3.**
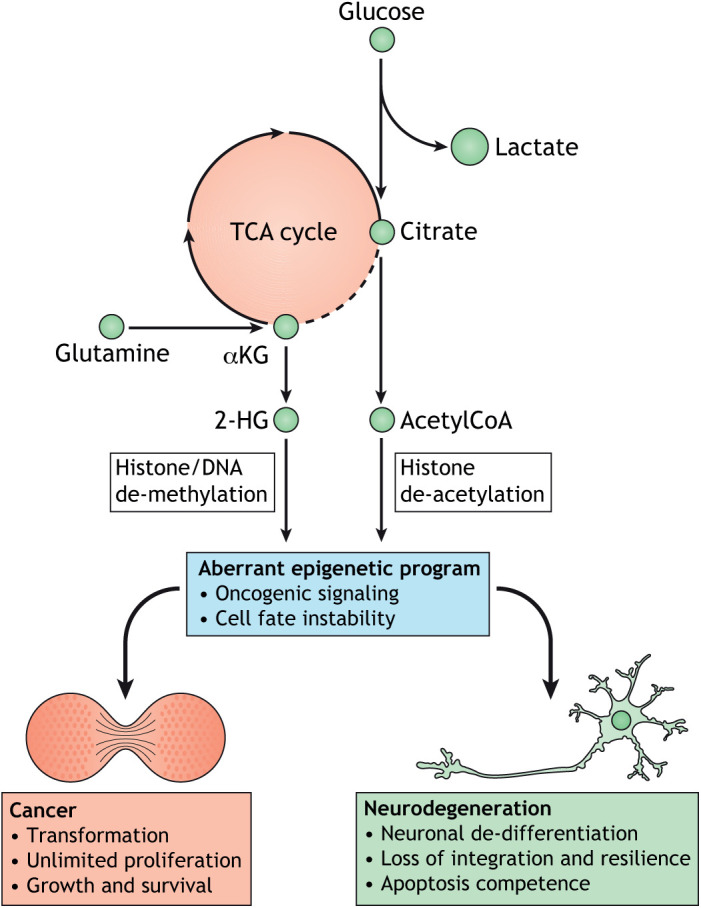
**Metabolic state shifts in cancer and neurodegeneration.** Glucose metabolism is severely altered in both cancer and neurodegeneration. Increased glucose uptake feeds lactate production and a flux to citrate is maintained. Citrate is mainly used for acetylCoA production and acetylation reactions, especially histone acetylation. Glutamine serves as an alternative carbon source to feed the TCA cycle and is metabolized to αKG and 2-HG to regulate histone and DNA methylation. The rewiring of metabolism in diseased cells is often so dramatic that it can be considered a complete metabolic state shift, which is a phenomenon otherwise only found during development. Consequentially, such shifts have large impacts on gene expression through epigenetic mechanisms, which further promote oncogenic signaling and cell fate instability. However, the effect of aberrant gene varies depending on the situation and cell type: in cancers, it leads to transformation, unlimited proliferation and growth; in neurons, it instead leads to neuronal de-differentiation, loss of integration of the neuronal network and resilience and apoptosis competence.

Metabolic flexibility is severely impaired during aging, which results in the inability to react adequately to environmental stressors. It plays an especially important role in neurons to adapt to rapid changes in energy demand. The basal metabolic state of neurons relies almost exclusively on oxidative phosphorylation, and, in comparison to other cell types, neurons show little tolerance to any attempts to alter this. Prolonged periods of synaptic firing, however, require large bursts of energy, triggering a switch to short-term glycolysis or an uptake of astroglial lactate (Mason, 2017; Mattson et al., 2008). This switch is only a short-term response to increased energy demands, and it is tolerable to neurons as they will quickly re-adapt their metabolism by recruiting mitochondria to synapses (Rossi and Pekkurnaz, 2019). During aging, neurons appear to lose this remnant of metabolic flexibility due to increasing demands in managing damage repair (Milanese et al., 2019). These neurons switch towards an age-associated ‘maintenance mode’ in which they direct glycolytic metabolites towards nucleotide production in the pentose phosphate pathway, and favor acetylCoA usage for histone acetylation (Fig. 3) (Milanese et al., 2019; Sivanand et al., 2017). Ultimately, this age-related rearrangement of the neuronal metabolic state reduces metabolic flexibility, which further de-stabilizes neuronal fate stability (Shetty et al., 2012).

### Neurodegeneration and metabolism

Aging is largely characterized by metabolic changes, and it is by far the biggest risk factor for the development and progression of neurodegenerative diseases (Hou et al., 2019). This even holds true for aggressive monogenetic forms of neurodegeneration. Here, we focus on the metabolic contributions to the two most common neurodegenerative diseases.

AD is the most frequent dementia, with over 90% of cases being sporadic and a small percentage caused by mutations in enzymes that constitute the amyloid precursor protein processing pathway. These so-called early onset familial forms of AD (fAD) appear earlier than sporadic forms, but still require decades to develop before the onset of symptoms (Shea et al., 2016). What distinguishes healthy aging from pathological aging is incompletely understood. Most knowledge regarding the mechanisms involved in AD is based on the amyloid hypothesis, which can be modeled with transgenic animal models and has led to the development of treatment strategies that inhibit plaque formation or remove plaques from AD brains (Elder et al., 2010). It is only within the past two decades that amyloid plaques have been detected to a greater degree in healthy old brains (Mehta et al., 2017; Mormino and Papp, 2018; Rodrigue et al., 2012). Further, in clinical trials we have seen anti-amyloid drugs failing to rescue cognition in advanced patients. Thus, contemporary views often entertain the hypothesis that neuronal cell death in AD is not primarily a response to the aberrant accumulation of external stressors, but rather an intrinsic pathogenic program of AD neurons that reduces neuronal resilience (Kole et al., 2013; Mertens et al., 2021). Age-related stressors, including the accumulation of amyloids, are then capable of triggering cell death specifically in AD neurons, leading to neurodegeneration (Kole et al., 2013; Tse and Herrup, 2017). This is especially interesting, because mature neurons of the CNS are known for having elaborate mechanisms to avoid cell death. In contrast to neuronal development, in which a balance of proliferation, apoptosis and differentiation is important for proper formation of the brain, neurons of the adult brain are required to maintain the complex neuronal network over a lifetime. Thus, mature neurons prioritize longevity and structural integrity over damage-induced cell death. This urges them to develop several anti-apoptotic brakes to avoid cell death, such as resistance to cytochrome c or a lack of apoptotic protease activating factor-1 (Apaf-1) expression (Kole et al., 2013).

So how can AD neurons circumvent this apoptotic brake and become competent for cell death? Recent transcriptional profiling of neurons directly converted from sporadic AD patients' fibroblasts and iPSC-derived neurons from fAD patients detected transcriptional and epigenetic signs of neuronal de-differentiation in AD towards a precursor-like state, upregulating genes involved in cell cycle and EMT (Caldwell et al., 2020; Mertens et al., 2021). Post-mortem brain tissue further showed signs of a metabolic state switch from oxidative phosphorylation to glycolysis in AD neurons (Marinaro et al., 2020 preprint). A loss of cell identity in neurons necessitates metabolic state instability and occurs in parallel with a loss of synaptic transmission and a gain of apoptotic competence (Kole et al., 2013; Marinaro et al., 2020 preprint). Despite the elaborate mechanisms of neurons to avoid cell death, a simple activation of glycolysis seems sufficient to trigger neuronal cell death (Herrero-Mendez et al., 2009). Considering neuronal development, a metabolic switch from oxidative phosphorylation to glycolysis could be interpreted as reverse differentiation, i.e. a return to immaturity. Remarkably, this phenomenon is excellently described in the cancer field, where such a switch is widely known as the Warburg effect (Box 1). However, whereas in cancer cells the Warburg effect supports the proliferative state of malignant cells, in neurons this same switch would likely lead to neuronal de-differentiation, perhaps even towards a point whereby the neurons become apoptosis competent again (Kole et al., 2013). This suggests that the metabolic switch in AD neurons might be an early regulator of de-differentiation (Fig. 3) (Su et al., 2016).

The living human brain is quite inaccessible to metabolic studies. Thus, similar to endeavors in psychiatric diagnostics research, aging- and neurodegeneration-associated biomarkers are being investigated in accessible tissues such as CSF or blood. In addition to serving as early diagnostic markers, metabolic alterations can be inferred from such data, albeit with a healthy amount of caution. Several biomarker studies have detected excess lactate concentrations in AD patient CSF, which might provide a hint towards increased glycolytic activity in the human brain during neurodegenerative diseases (Henchcliffe et al., 2008; Liguori et al., 2016; Sonntag et al., 2017; Szelechowski et al., 2018). However, the neuronal and potentially non-neuronal cell sources that evoke these changes remain obscure from such data. The upregulation of glycolysis might be a compensatory effect in response to an energy crisis, or it might represent a pathological program similar to what is observed in age-related cancers (Marinaro et al., 2020 preprint). Recent studies showed that primary mouse astrocytes carrying the *APOE4* risk allele switch to aerobic glycolysis and possess a more limited metabolic flexibility (Farmer et al., 2020 preprint; Williams et al., 2020). Decreased metabolic support of APOE4 astrocytes for neurons might force neurons to increase basic metabolic activity, which would render neurons inflexible and unable to respond to metabolically demanding processes, such as synaptic activity, DNA damage repair or proteostasis (Domise et al., 2019; Qi et al., 2021; Ramamurthy et al., 2014; Szelechowski et al., 2018). The accumulation of certain metabolites, such as acetylCoA or αKG, further destabilizes cell identity by changing the epigenetic landscape (Benito et al., 2015; Huo et al., 2019; Nativio et al., 2018; Schueller et al., 2020; Watson et al., 2016). All in all, evidence suggests a reversal to a precursor-like state that is accompanied by a metabolic switch, rendering neurons susceptible to cell death. Further, AD-associated metabolic changes could be found in amino acid and lipid metabolism (de Leeuw et al., 2017; Trushina et al., 2013). Changes in amino acid concentrations suggest aberrant neurotransmitter production and usage in human AD brains, which would be consistent with the well-described changes preceding neurodegeneration in animal models of AD (Selkoe, 2002; Trushina et al., 2013). Positron emission tomography (PET) imaging is widely used to visualize glucose consumption in certain brain areas during neurodegeneration. Synaptic loss and neuronal de-differentiation are associated with increased neuronal death in affected brain areas, whereas glial cells are mainly unaffected (Marinaro et al., 2020 preprint). AD patients show glucose hypometabolism in highly affected brain areas, which is correlated with disease progression (Schöll et al., 2011; Hammond et al., 2020). For example, in carriers of the *APOE4* allele, which have a slightly increased risk for developing AD, PET imaging and plasma metabolomics have detected a glucose hypometabolism decades before the putative onset of AD (Farmer et al., 2020 preprint; Reiman et al., 1996). PET imaging thus provides unique insights into patients' brains, and helps to identify severely affected brain regions, but does not allow one to draw conclusions about cell type-specific alterations in glucose metabolism. Current evidence might be indicative of an early neuronal metabolic switch towards glucose utilization and alterations in amino acid and lipid metabolism in AD patients, all of which precede the onset of dementia symptoms, and thus might provide new diagnostic tools and options for intervention.

Parkinson's disease (PD) is characterized by severe motor deficits and dementia caused by a loss of dopaminergic neurons in the substantia nigra. Familial PD can be caused by mutations in mitochondrial proteins, directly linking metabolism to neurodegeneration in PD. Mutations in parkin and *PINK1*, genes encoding two proteins involved in mitochondrial quality control, are the most common autosomal recessive mutations causing the loss of dopaminergic neurons in PD. Exposure to pesticides that inhibit mitochondrial function further contributes to the development of sporadic PD (Park et al., 2018). The accumulation of damage, together with the physiological decline of metabolic functions with age, contributes to the late onset of neurodegenerative disease. For example, decrease in amino acid homeostasis and mitochondrial hyperactivity precede the loss of function of mitochondrial complex I observed in post-mortem brain tissues (Mor et al., 2020). Serum and plasma metabolomics of PD patients with different mutational backgrounds further shows dysfunctional mitochondrial activity. Both glucose and fatty acid oxidation are impaired in PD (Ahmed et al., 2009; Okuzumi et al., 2019). Additionally, neuroinflammation further accelerates PD progression (Martin-Ruiz et al., 2020). Evidence indicates that glial cells, and also neurons, can enter a senescence-like state, and that senescent dopaminergic neurons accumulate during aging and might contribute to PD progression via dysfunction and the senescence-associated secretory phenotype (SASP) (Box 2) (Martínez-Cué and Rueda, 2020; Riessland et al., 2019; Saez-Atienzar and Masliah, 2020). Certainly, however, an increase in activated microglia can already be observed before the onset of symptoms in PD, and may contribute to the death of dopaminergic neurons. The role of microglia, and their regulation by metabolites, has been best studied in multiple sclerosis. This inflammatory disease of the brain is characterized by demyelination and axonal damage, and was formerly considered to be an auto-immune disease. Contrary to this hypothesis, it is now understood that metabolic dysregulation in microglia leads to aberrant activation and secretion of immunomodulatory metabolites (De Riccardis et al., 2015; La Rocca et al., 2017; Peruzzotti-Jametti et al., 2018). These inflammatory signals further inhibit oligodendrocyte differentiation, which are required for re-myelination of lesions (Domingues et al., 2016).

**Box 2. The anti-tumor metabolic state of senescence**

Chronic and irreparable damage that is not compatible with normal cellular physiology, and that might cause oncogenic transformation, typically leads to programmed cell death, or to senescence. Senescence is defined as the permanent arrest of the proliferative state of the cell, and entering senescence entails many cellular changes that are also relevant to post-mitotic cells, including excitatory changes and a metabolic switch (Kuilman et al., 2010). One cell cycle-independent feature is the senescence-associated secretory phenotype (SASP), which describes the release of pro-inflammatory molecules from senescent cells (Sabbatinelli et al., 2019). The SASP is believed to attract immune cells to scavenge and degrade damaged cells. An age-associated accumulation of senescent cells, however, blocks the anti-tumor effects of senescence, and supports malignant transformation (Liu et al., 2018). This might be due to more cells turning senescent in old age or an age-exhausted immune system. Interestingly, the senescence state has its own, somewhat unexpected, metabolic state. Even though growth is inhibited in senescent cells, they are metabolically very active. The senescent metabolic state is characterized by an increase in both glycolysis and mitochondrial oxidation, which is thought to be necessary for the massive and desperate production and release of SASP factors. Interestingly, although the end-product in cancer and neurodegeneration differs greatly, neural cell senescence might be a causative factor for age- and disease-related neuroinflammation (Riessland et al., 2019; Saez-Atienzar and Masliah, 2020). This is also supported by the metabolism-centric observation of the same initial mechanisms in both cancer and neurodegeneration. Similar to cancer cells, data from human cerebrospinal fluid studies, post-mortem brain samples and patient-derived cell models indicate that neurons partially lose their grip on their differentiated identity in several neurodegenerative diseases, including Alzheimer's disease, amyotrophic lateral sclerosis, Huntington's disease and other forms of neurodegeneration (Atlante et al., 2017; Sonntag et al., 2017; Vallée et al., 2018; Arendt et al., 2000). However, on the population level, an inverse relationship between cancer and neurodegeneration exists, meaning that patients suffering from cancer are less probable to develop neurodegeneration and vice versa, and it remains to be fully determined to what extent senescence is a cause, or merely a byproduct, of neurodegeneration (Sánchez-Valle et al., 2017).

In summary, changes in metabolic genes can cause late-onset neurodegeneration, and age- and disease-related metabolic state switches put neural cell-type identity under siege, which leads to a loss of resilience of neural cells and their eventual degeneration. A better understanding of age-related metabolic events might lead to early diagnostics and treatments for neurodegenerative diseases and might further help to control neuroinflammation.

## Conclusion and perspective

There is robust evidence suggesting that metabolic states and individual metabolites play an essential role in cell fate decisions beyond their functions in adapting to energy demands (Ando et al., 2019; Tran et al., 2019; Zhou et al., 2019). Orchestrated changes in the wiring of metabolic pathways and switches between defined metabolic states have proven essential for cell fate determination during development, and to maintain the proper homeostasis and function of the adult human brain. Individual metabolites have been identified and have demonstrated to fulfil essential and instructive roles in the epigenetic landscape, thus controlling cell identity, function and resilience. Even slight imbalances in certain metabolites can lead to severe cellular changes and dysfunction, not only during development. Further, complete metabolic shifts between defined metabolic states constitute important upstream components of the pathology of several diseases, including cancer and age-related neurodegeneration. In contrast to most somatic cells however, which respond to pathogenic metabolic state shifts with malignant transformation, neurons instead appear to lose their outstanding resilience and fate stability and re-activate the cell death mechanism. Thus, much new data render neural cell metabolism research an exciting topic to better understand the physiology and pathophysiology of the human brain.
